# Experimental microembolism induces localized neuritic pathology in guinea pig cerebrum

**DOI:** 10.18632/oncotarget.3599

**Published:** 2015-03-14

**Authors:** Jian-Ming Li, Yan Cai, Fei Liu, La Yang, Xia Hu, Peter R. Patrylo, Huaibin Cai, Xue-Gang Luo, Dong Xiao, Xiao-Xin Yan

**Affiliations:** ^1^ Department of Anatomy and Neurobiology, Central South University School of Basic Medical Science, Changsha, Hunan, China; ^2^ Neuroscience Research Center, Changsha Medical University, Changsha, Hunan, China; ^3^ Department of Neurosurgery, The Third Xiangya Hospital, Central South University, Changsha, Hunan, China; ^4^ Center for Integrated Research in Cognitive and Neural Sciences, Southern Illinois University School of Medicine, Carbondale, Illinois, USA; ^5^ Laboratory of Neurogenetics, National Institute on Aging, Bethesda, Maryland, USA; ^6^ State Key Laboratory of Coal Resources and Safe Mining, China University of Mining and Technology, Xuzhou, Jiangsu, China

**Keywords:** Alzheimer's disease, amyloid pathology, axonal pathology, brain aging, silent stroke

## Abstract

Microbleeds are a common finding in aged human brains. In Alzheimer's disease (AD), neuritic plaques composed of β-amyloid (Aβ) deposits and dystrophic neurites occur frequently around cerebral vasculature, raising a compelling question as to whether, and if so, how, microvascular abnormality and amyloid/neuritic pathology might be causally related. Here we used a guinea pig model of cerebral microembolism to explore a potential inductive effect of vascular injury on neuritic and amyloid pathogenesis. Brains were examined 7-30 days after experimental microvascular embolization occupying ~0.5% of total cortical area. Compared to sham-operated controls, glial fibrillary acidic protein immunoreactivity was increased in the embolized cerebrum, evidently around intracortical vasculature. Swollen/sprouting neurites exhibiting increased reactivity of nicotinamide adenine dinucleotide phosphate diaphorase, parvalbumin, vesicular glutamate transporter 1 and choline acetyltransferase appeared locally in the embolized brains in proximity to intracortical vasculature. The embolization-induced swollen/sprouting neurites were also robustly immunoreactive for β-amyloid precursor protein and β-secretase-1, the substrate and initiating enzyme for Aβ genesis. These experimental data suggest that microvascular injury can induce multisystem neuritic pathology associated with an enhanced amyloidogenic potential in wild-type mammalian brain.

## INTRODUCTION

Amongst many competing etiological theories, the vascular hypothesis posts chronic cerebrovascular deficits as a causal factor for Alzheimer's disease (AD) [1-9]. This hypothesis appears coherent from a number of epidemiological, pathological and clinical perspectives, such as (1) Aging, the best known cause of AD, is associated with vascular hardening, fragility and vulnerability to injury, as evidenced by an increased prevalence of cardiovascular diseases among the elderly [10, 11]; (2) Many cardiovascular or metabolic disorders with cardiovascular comorbidity (e.g., atherosclerosis, hypertension, hyperlipidemia, diabetes) are known risk factors for AD [12-14]; (3) Postmortem and antemortem imaging studies converge to show frequent occurrence of brain microbleeds among the elderly and individuals with mild cognitive impairment or symptomatic dementia [15-17]; (4) Vascular and AD-type dementias are often difficult to differentiate clinically because of the many shared neuropathological and neurological manifestations [18]. Thus, more basic research regarding vascular risk factors relative to AD-type neuropathology is warranted, which could be informative for preventing dementia among the elderly [19-21].

Neuritic amyloid plaques are a hallmark neuropathology of AD and may occur in the brain of non-demented elderly as well [22]. This lesion consists of localized extracellular β-amyloid (Aβ) deposition in association with dystrophic neurites [23, 24]. The amyloid deposits are insoluble aggregates of mixed forms of Aβ peptides derived from proteolytic processing of the β-amyloid precursor protein (APP) by β-secretase-1 (BACE1) and γ-secretase complex [25, 26]. In transgenic AD models increased BACE1 expression inherent with neuritic dystrophy occurs along with Aβ deposition [27-29]. The causal relationship between Aβ deposition and the formation of dystrophic neurites in the human brain remains a topic of discussion [30-32].

An anatomical association between amyloid plaques and cerebral vasculature has been shown in postmortem human brain for several decades [33, 34], with some studies showing clear evidence of hemorrhage around plaques [35-38]. Further, modern imaging studies have demonstrated a link between small arterial changes and Aβ deposition in the living brain of dementia patients [39]. Thus, vascular dysfunction (e.g., hypoperfusion) and/or structural damage (e.g., leakage) are considered to potentiate amyloid pathology [1, 2, 37, 38]. In a transgenic model of AD, focal photothrombotic lesion induces transient plaque formation likely due to impaired Aβ clearance [40]. In adult rats, penetrating cerebral injury results in hemorrhage and transient upregulation of Aβ and APP along the needle track [41]. In support of vascular leakage playing a role in plaque development, some blood proteins, including hemoglobin and apolipoproteins, are shown to promote Aβ aggregation [42, 43].

A preferential distribution of BACE1 containing dystrophic neurites in proximity to cerebral microvasculature has been recently established in the brain of aged nonhuman primates and AD human subjects [44]. Here we attempted to explore whether localized microvascular injury might promote amyloidogenic neuritic pathology, and perhaps, plaque formation, in a wild-type animal model. Using a histologically verifiable approach that causes clear focal vascular microembolism, we here report histopathological findings that experimentally induced microvascular injury in the guinea pig cerebrum can lead to localized formation of dystrophic neurites, with some exhibiting strong APP and BACE1 labeling suggestive of an enhanced amyloidogenic potential.

## RESULTS

### Assessment of dose-effect on experimental microembolism in the cerebral neocortex

**Figure 1:**
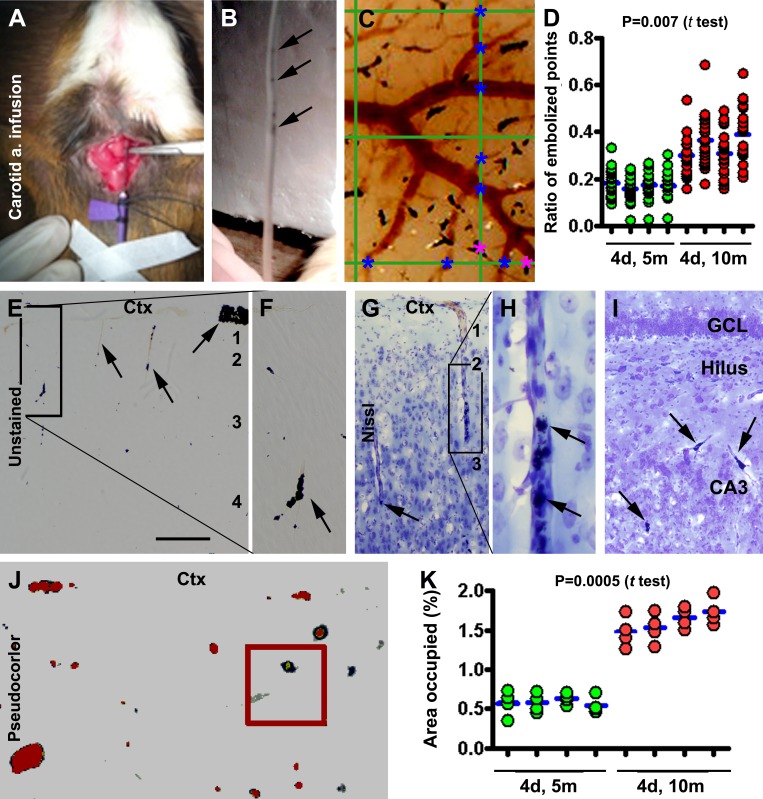
Modeling and characterization of cerebral microembolism in guinea pigs Panels (A, B) show the method for common carotid artery infusion of sterile coal particles (pointed by arrows in the infusion tube in B). Panel (C) illustrates a method for quantifying the embolized (marked with purple asterisks) and non-embolized (marked with blue asterisks) pial arterial intersections at the vertical and horizontal lines (10 for each) of a measuring template (10×10 grids) placed over the image of the dorsal cerebral surface. Panel (D) plots the ratios of the embolized to total pial arterial sites obtained from animals (n=4/group) surviving 4 days (4d) following infusion for 5 minutes (5m) and 10 minutes (10m). For a giving animal, each red dot represents a ratio obtained from a horizontal or vertical line of the measuring grid, with the blue bar showing the mean ratio for the brain. The mean ratio is significantly greater in the 10 than 5 minute infusion groups (P=0.007, paired Student *t*-test). Panels (E-I) show localized embolization of pial and intracortical arterioles (examples pointed by arrows) seen in brain sections before (E, F) and after Nissl stain (G-I) in the cortex (Ctx) and hippocampal formation (I). The perivascular space appears separated from surrounding neural tissue around the embolized vessels (G-I). Panel (J) shows a method to quantify the area occupied by the embolized sites in cerebral sections with threshold-based automatic selection. Panel (K) plots the data obtained over the parietotemporal cortex in 4 sections passing the anterior commissure, the mid-septum, the anterior end of hippocampus and the temporal pole. The mean is significantly greater in the 10 relative to 5 minute infusion group. Arab numbers indicate the cortical layers. GCL: granule cell layer. Scale bar=200 μm in (E), equal to 100 μm for (F, G, I), 50 μm for (H, J).

During a series of pilot experiments, we learned that coal particles, 50-100 mesh-size at 1% concentration, were suited for inducing microvascular embolization in a manner that allowed for survival studies (Fig. 1A, B). Infusing particles <50 mesh-size required the use of larger needles and increased mortality subsequent to poor artery recovery and postoperative bleeding. Infusing particles >100 mesh-size were less effective in causing microembolism, likely due to the particles filtering-through with the circulation. Most animals could recover post-surgery when infusions lasted from 5 to 10 minutes. However, by the end of the first week following infusion, mortality was high (up to 80%) among animals with the longer infusion times. Among animals that received a 5 minute infusion, the mortality was relatively low (~15%, counting all of the animals that recovered from surgery and could survive for 1 month).

To understand the dose-effect relationship, we compared the extent and pattern of the experimentally induced vascular embolization in animals surviving 4 days with 5 relative to 10 minute infusion by quantitative analysis of embolized pial vascular sites on the dorsal brain surface (Fig. 1C, D), as well as intracortical vascular sites in coronal cerebral sections (Fig. 1E-J). The mean ratio of embolized to total pial arterial points intersecting at the dividing lines of a standard measuring grid (10×10 lines, placed on both hemispheres) was 0.17 ± 0.01 and 0.34 ± 0.04 for the animal groups that received 5 versus 10 minutes of infusion (Fig. 1D) (P= 0.008, by paired Student *t* test). In coronal brain sections, the embolized sites occurred discretely but throughout the forebrain structures including the cortex, hippocampal formation and striatum in both hemispheres, involving largely small pial and intracortical arterioles (Fig. 1E-I). In Nissl stained sections, the perivascular space at the embolized arterioles tended to increase (Fig. 1G-I). The extent of embolization to intracortical arteries was quantified in each brain over the parietotemporal region. The mean area occupied by the embolized vessels (% of the total cortical grey and white matter area) was 0.59 ± 0.17 % and 1.66 ± 0.36 % for the animals received 5 and 10 minute infusion, respectively (Fig. 1J, K), with a significant difference between the two groups (P= 0.003, by paired Student t test).

### Glial activation in response to experimental microembolism

The embolized coal particles remained visible for 7-10 days post-infusion (based on examination of brains collected without perfusion). Brains from animals surviving 7, 14 and 30 days were studied following fixation via cardiovascular perfusion to allow optimal immunohistochemical staining. An inflammatory response of astrocytes was prominent in the embolized brains relative to sham-operated controls. Thus, enhanced glial fibrillary acidic protein (GFAP) immunoreactivity (IR) was observed in animals surviving 7-30 days, with morphological and densitometric data from the 14 day surviving groups presented below.

**Figure 2:**
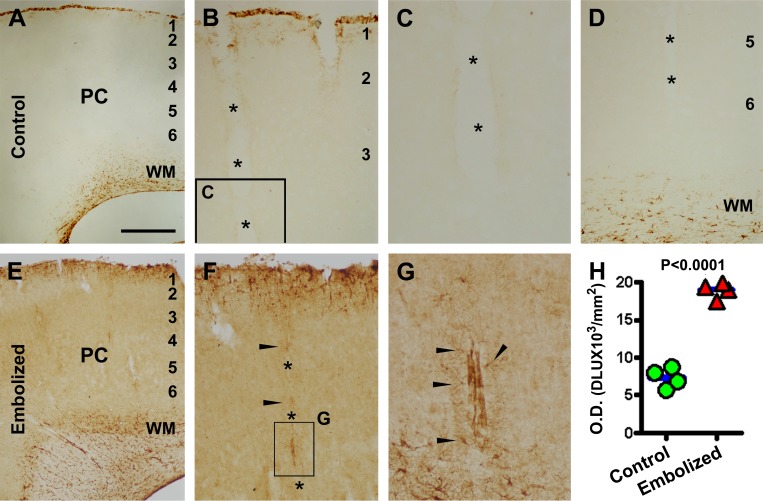
Representative images and densitometry showing increased glial fibrillary acidic protein (GFAP) immunoreactivity (IR) in the embolized cerebral cortex relative to control Panels (A-D) illustrate GFAP IR in the parietal cortex (PC) at the level of mid-septum from a sham-operated animal, with the labeling occurring largely at the pia and in the white matter (A, B, D). Faint labeling exists around vessels (marked with *) invading the cortex (B-D). Panels (E-G) illustrate GFAP IR in a comparable region of the cortex from an embolized animal, which is overtly increased relative to control. Note the heavy GFAP labeling in astrocytic cells around intracortical vessels (F, G, arrows). Panel (H) plots the mean optic densities in 4 sham-operated and 4 embolized animals surviving 14 days, showing significant difference between the two groups. The mean density for each brain is calculated based on densitometry over the parietotemporal region in sections at 4 comparable rostrocaudal levels. Scale bar=200 μm in (A) applying to (E), equal to 100 m for (B, D, F), 50 μm for (C) and 25 μm for (G).

In control animals, GFAP IR was largely seen around the pia and white matter (Fig. 2A). At higher magnification, GFAP labeled elements were packed along the pial surface with a few cells resembling astrocytes in the upper portion of layer I, and faintly labeled cells or processes lining along a few vessels invading the cortex (Fig. 2B, C). GFAP immunoreactive astrocytic cells in the white matter occurred predominantly close to the lateral ventricle (Fig. 2A, B). In the embolized brains, enhanced GFAP IR occurred throughout layer I (Fig. 2E, F), along many vertically arranged intracortical vessels (Fig. 2F, G), and over the entire white matter (Fig. 2A). A densitometry of layers I to VI in the parietotemporal neocortex indicated that the mean density of GFAP IR was greater (P=0.002, by paired Student-*t* test) in the embolized group (7333±1305 DLU/mm^2^) compared to control (18982±1057 DLU/mm^2^) (Fig. 2H).

### Neuritic alterations in response to experimental microembolism

**Figure 3:**
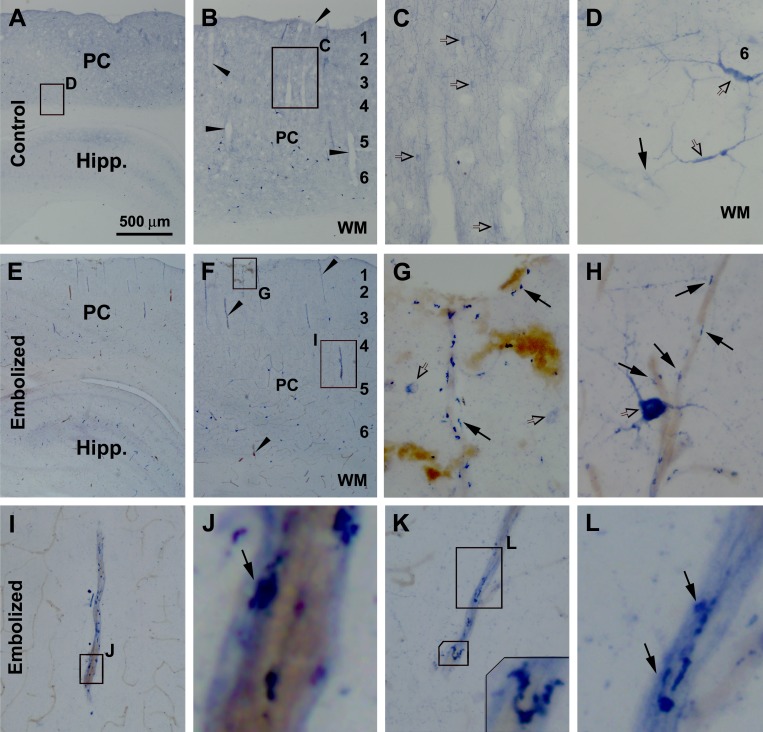
Early sprouting of nicotinamide adenine dinucleotide phosphate diaphorase (NADPH-d) positive neuronal processes around embolized cerebral vasculature Shown are images of the parietal cortex (PC) and hippocampal formation (Hipp) in sham-operated (A-D) and embolized (E-L) animals surviving 7 days. In the control brain, intracortical vessels are surrounded by even and fine NADPH-d positive processes (C, D). In the embolized brain, increased NADPH-d reactivity is visible around blood vessels at low magnification in the cortex and white matter (WM) (E, F), with swollen terminal endings with heavy NADPH-d reactivity clearly seen at high magnifications (G-L). The labeled swollen perivascular neuritic elements are irregular in shape and tended to align along blood vessel longitudinally (G-L). Enlarged panels and framed areas are as indicated. Arrowheads point to examples of intracortical vessels. Arrows point to NADPH-d positive processes and terminal endings apposing to blood vessels. Open arrows point to examples of type I (D, H) and type II (C, G) NADPH-d positive neurons. Scale bar=500 μm in (A) applying to (E), equal to 200 μm for (B, E), 50 μm for (C, I), 25 μm for (D, G, H, K), 10 μm for (L) and 5 μm for (J).

**Figure 4:**
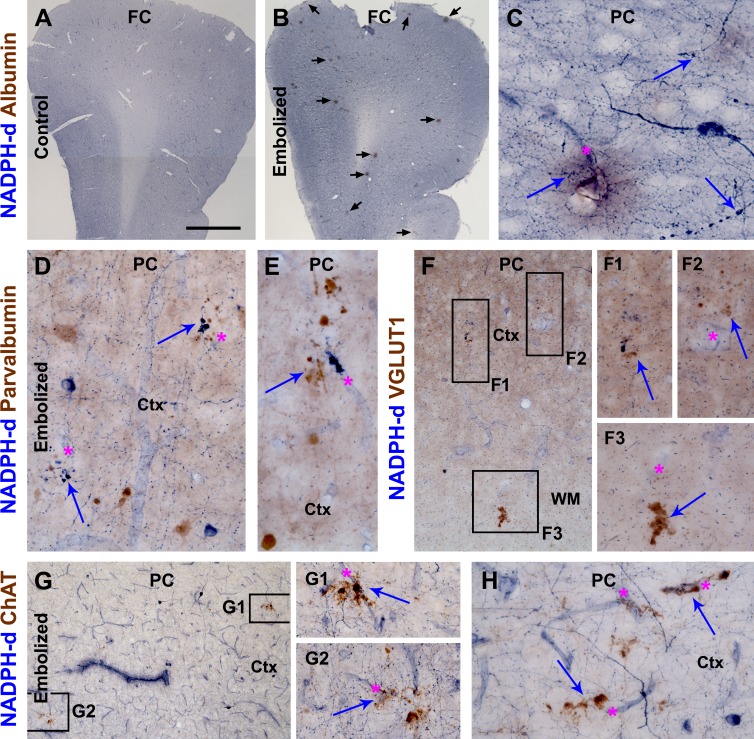
Cerebral vascular injury and neuritic pathology in guinea pigs surviving 30 days post embolization Panels (A, B) are low power views of nicotinamide adenine dinucleotide phosphate diaphorase (NADPH-d) reactivity counterstained immunohistochemically for guinea pig albumin in the frontal cortex (FC) from a control (A) and an embolized animal (B). Note the localized areas (pointed by arrows in B) with brown DAB reaction products representing vascular leakage of albumin. Images of other panels are taken from the parietal cortex (PC). Panel (C) is a high magnification view showing diffuse albumin IR around a vessel with some histological disruption, with arrows pointing to swollen NADPH-d processes. Panels (D, F) show paravascular swollen/sprouting neurites (arrows) immunolabeled for parvalbumin. Panel (F) and enlargements (F1-3) show swollen neurites (arrows) immunoreactive for vesicular glutamate transporter-1 (VGLUT1) in the cortex (Ctx) and white matter (WM) around blood vessels (asterisks) in the embolized brain. Similarly, swollen neurites around blood vessels may express choline acetyltransferase (ChAT) (G, G1, G2, H). Notably, swollen neurites labeled by NADPH-d and another neuronal marker may co-exist around the same vascular site (D, E, F1, G1). Scale bar=500 μm in (A) applying to (B), equal to 100 μm for (D, G) and 50 μm for (C, D1-3, E, F, G1, G2, H).

Nicotinamide adenine dinucleotide phosphate diaphorase (NADPH-d) histochemistry can extensively label the processes of neurons producing nitric oxide [45, 46]. NADPH-d histochemistry and immunohistochemistry for several other neuronal markers were carried out (by batch processing of sections from embolized and control brains). Enhanced NADPH-d reactivity was visible around cerebral vasculature in the embolized relative to control brains from animals surviving 7 (Fig. 3) and 14 days (not shown), and were especially prominent by 30 days (Fig. 4). In sham-operated brains, NADPH-d reactive type I and type II interneurons were present in the cortex and subcortical white matter [46], with fine processes distributed extensively in the grey matter (Fig. 3A-D). Overall, no differences could be identified with regard to the amount, morphology and labeling intensity between NADPH-d labeled processes apposing to and distant from intracortical blood vessels (Fig. 3C, D). At some locations, NADPH-d reactive processes were seen to run towards blood vessels (Fig. 3D). Compared to controls, increased NADPH-d labeling was readily visible in the embolized brains at 7 days post-surgery (Fig. 3E, F). At higher magnification, terminal endings with heavy NADPH-d reactivity occurred at blood vessels entering the cortex from the pia (Fig. 3F, G) as well as those in the cortical parenchyma (Fig. 3H-L). The labeling intensity of these terminal profiles was apparently increased relative to those distally located from the blood vessels in the embolized brains (Fig. 3G, H, I, K), or in the processes in the control brains (Fig. 3C, D). The swollen perivascular neritic elements were irregular in shape and tended to align along a vessel longitudinally (Fig. 3H, J, L).

As NADPH-d histochemistry can visualize the outline of blood vessels [45], dual histochemical (NADPH-d) and immunohistochemical (other markers) staining was used to explore neuritic pathology relative to vasculature, using sections from animals surviving 14 (data not shown) and 30 (Fig. 4) days. Vascular leakage was additionally verified using an antibody specific to guinea pig albumin, which showed no labeling in sham-operated control brains (Fig. 4A-C). At high magnification, albumin IR was associated histologically with localized vascular disruption and swollen NADPH-d positive processes, and appeared to diffuse into the paravascular parenchyma (Fig. 4C). Localized swollen neurites immunoreactive for parvalbumin, another excellent marker of GABAergic interneurons, were found around intracortical blood vessels (Fig. 4D, E). Swollen and sprouting processes immunoreactive for vesicular glutamate transporter-1 (VGLUT-1), a marker for glutamatergic presynaptic terminals, were found in the grey and white matter (Fig. 4F). Moreover, swollen/sprouting neurites immunolabeled for choline acetyltransferase (ChAT), a marker for cholinergic neurons and terminals, were observed in the embolized brains around vascular profiles (Fig. 4G, H). Notably, in many cases NADPH-d positive swollen neurites were found to co-exist with GABAergic (Fig. 4D, E) glutamatergic (Fig. 4F), and cholinergic (Fig. 4G1) dystrophic-like terminals around the same locations.

### Increased labeling for amyloidogenic proteins in response to experimental microembolism

**Figure 5:**
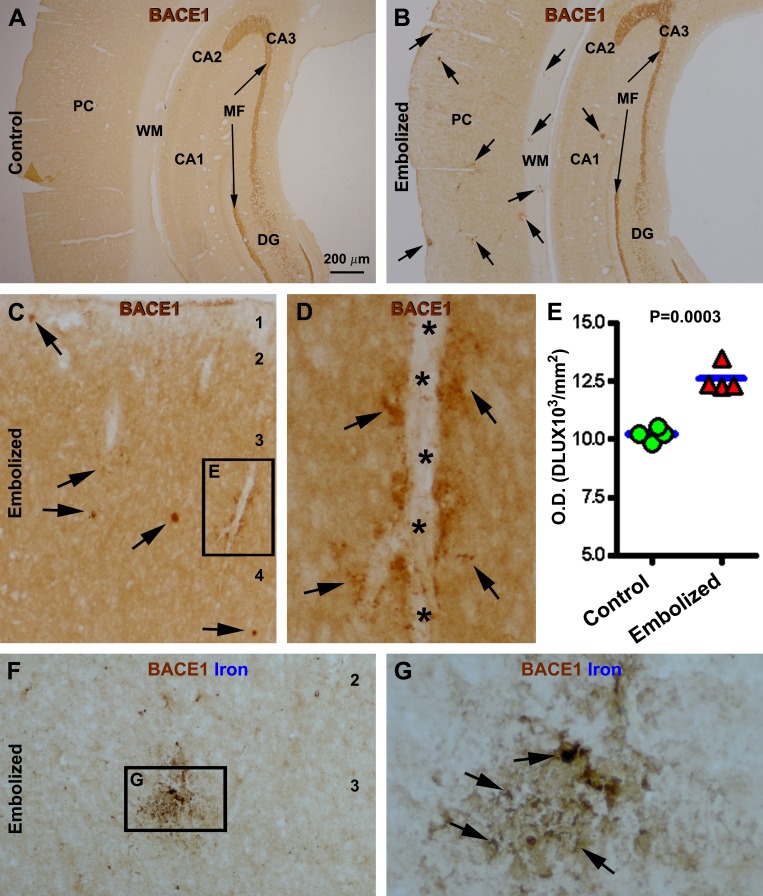
Localized increase of β-secretase-1 (BACE1) immunoreactivity (IR) in embolized cerebrum relative to sham-operated control 14 days post operation Panel (A) shows a neuropil labeling pattern of BACE1 IR over parietal cortex in a control animal, with strong IR at the mossy fiber (MF) terminal field. In comparison, BACE1 IR is locally increased (pointed by arrows) over the background in the cortex and white matter in the embolized cerebrum, while the mossy fiber labeling is comparable relative to control (B). Panel (C, D) show high power views of the localized increase of BACE1 IR, with some profiles (arrows) clearly occurring along intracortical vessels. Panel (E) plots the mean optic densities of BACE1 IR from 4 control and 4 embolized animals, with significant difference between the two groups. Panels (F, G) show BACE1 labeling with Perl's Prussian blue counterstain. Note the ferric iron deposition (arrows) at an area with locally increased BACE1 IR (G). Arab numbers indicate cortical layers. PC: parietal cortex; CA1-3: hippocampal CA sectors; DG: dentate gyrus. Scale bar=200 μm in (A) applying to (B), equal to 50 μm for (C, F) and 12.5 μm for (D, G).

Compared to controls, increased BACE1 IR was observed in the embolized brains from animals surviving 7 (not shown), 14 and 30 days, and was more evident in the latter two groups. Thus, in control brains, BACE1 IR exhibited a normal pattern of distribution, with labeling appearing as a diffuse neuropil-like reaction across the cortical grey matter and hippocampal laminas, with a distinct labeling at the mossy fiber terminal field (Fig. 5A). In contrast, at low magnification, BACE1 IR was locally increased (relative to baseline density) in the neocortex and hippocampal formation of embolized brains, in both the grey and white matter areas, and was often seen around vascular profiles (Fig. 5B). At higher magnification, many of the BACE1 labeled perivascular profiles appeared as swollen sphericles and neuritic processes (Fig. 5C, D). Densitometry performed on the parietotemporal neocortical region of brains from animals surviving 14 days confirmed a significant increase of overall BACE1 IR in the embolized group (12594±585 DLU/mm^2^) compared to control (10208±276 DLU/mm^2^) (P=0.003, by paired Student-*t* test) (Fig. 5E). To assess the spatial relationship of increased BACE1 IR and vascular injury, immunostained sections were counterstained with Perl's Prussian blue to visualize ferric iron deposition. With the DAB-reaction background, iron deposition (originally blue) appeared grey/black and co-occurred in areas with increased BACE1 IR; some neuritic profiles displayed co-staining (Fig. 5F, G).

**Figure 6:**
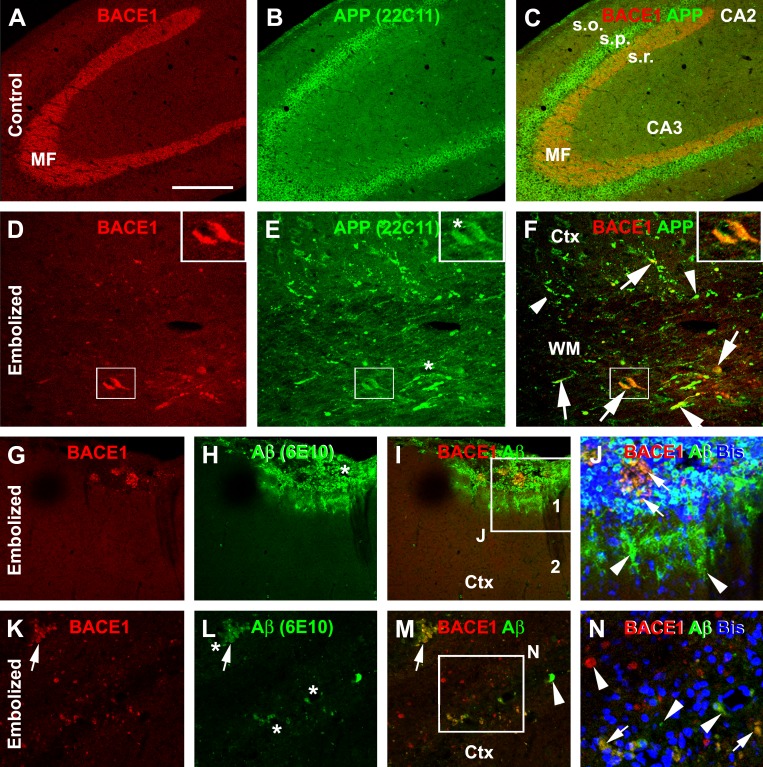
Confocal double immunofluorescent characterization of amyloidogenic proteins in the cerebrum of embolized relative to control guinea pigs surviving 30 days Panels (A-C) show a normal distribution pattern of β-secretase-1 (BACE1) and β-amyloid precursor protein (APP) immunoreactivity (IR) in the hippocampal formation in a control brain, with distinct BACE1 IR at the mossy fiber (MF) terminals and APP IR in the somata of CA3 pyramidal neurons. Panels (D-F) show a partial colocalization of BACE1 and APP IR in swollen neurites in the cortex (Ctx) and white matter (WM) in the embolized cerebrum. Panel (G-J) show a part colocalization of BACE1 and 6E10 IR at a large profile around the pia, with local histological distortion. Note the cloudy extracellular 6E10 IR at the periphery of the lesion site (J). Panel (K-N) show BACE1 and 6E10 labeled neuritic sphericles in the cortical grey matter, exhibiting a partial colocalization. Arrows point to examples of profiles with colocalization, while arrowheads point to examples of profiles labeled by only one marker. Enlargements of framed areas are as indicated. Arab numbers denote cortical layers. Other abbreviations: s.o.: stratum oriens; s.p.: stratum pyramidale; s.r.: stratum radiatum. Scale bar=200 μm in (A) applying to (B, C), equal to 50 μm for (D-F, G-I, K-M) and 25 μm for (J, N).

To determine the occurrence of increased BACE1 IR in dystrophic neurites and its relevance to other Aβ related markers, confocal double immunofluorescence for BACE1/APP and BACE1/6E10 was carried out in embolized and control brains from animals surviving 30 days (Fig. 6). In control brains, neither BACE1 nor APP antibody labeling was observed in neuritic processes in the grey and white matter of the cortex (not shown) or hippocampal formation (Fig. 6A-C). In contrast, swollen neurites expressing APP were clearly present in these regions in the embolized brains, especially in the white matter (where no any neuritic labeling was seen in controls) (Fig. 6D-F). Likely because APP is a robust marker for axonal pathology [47], the increased BACE1 IR was colocalized in a subset of APP labeled swollen neurites (Fig. 6A-F).

6E10 is a monoclonal antibody raised against the N-terminal region of human Aβ, which could label various Aβ products, with a potential cross-reactivity to APP and/or its immediate BACE1 cleavage products, i.e., the β-C-terminal fragments (β-CTF) [27]. A partial colocalization pattern was observed in BACE1 and 6E10 double immunofluorescence. Thus, in areas with relatively severe vascular/histological alterations, including around the pia, a large amount of 6E10 IR was seen to accompany some smaller BACE1 labeled profiles (Fig. 6G-J). Among these sites, diffuse 6E10 IR could be found in the periphery of the lesion site, showing a cloudy pattern likely suggestive of Aβ presence in extracellular space (Fig. 6J). Many neuritic elements in the cortex and white matter exhibited increased BACE1 IR, with some arranged as clusters while others occurred in isolation. A subset of these BACE1 labeled neurites showed 6E10 immunofluorescence (Fig. 6K-N). There were also some 6E10 labeled profiles that did not exhibit clear BACE1 immunofluorescence (Fig. 6M, N).

## DISCUSSION

### Experimental microembolism induces localized vascular injury

AD and vascular dementia are largely sporadic in nature; wild-type animal models could be useful to explore relevant pathogenic issues. Guinea pigs are used in the present study because of a technical feasibility (i.e., the carotid artery of rats/mice was found to be too small and difficult to perform infusion in our pilot study) as well as a consideration that these animals express the human-type APP [48]. Experimental cerebral microembolism induced by the carbon-nature artificial emboli can cause significant acute/sub-acute impact on animal survival. Blockage of cerebral microvasculature with a total embolized size over 1% of the cortical area resulted in a great mortality within a few days post operation. The impact of acute diffuse cerebral microembolism on rodents is not well characterized. According to literature, mice and rats could survive a great extent of regional brain traumatization (e.g., the stroke model by ligation of the entire middle cerebral artery). We do not yet know whether guinea pigs are more (relative to mice or rats) sensitive to the blockage of cerebral blood supply in general or to the procedure of diffuse microembolism used in the present study in specific. Nonetheless, the current study demonstrates that localized embolization occupying about or less 0.5% of total cortical area may allow surviving studies. Under such an experimental paradigm, unilateral common carotid artery infusion resulted in embolization of largely small pial and intracortical arteries, associated with vascular disruption and destruction as well as localized leakage of blood components, as indicated by extravascular albumin and iron deposition. Importantly, the experimentally induced microembolism can trigger histologically evident glial and neuronal responses.

### Perivascular glial and neuritic elements respond to microembolism

Glial and neuronal processes apposing microvasculature comprise an integral part of the neurovascular unit, a fundamentally important system for normal brain function. Vascular, including microvascular, injuries can induce glial activation and inflammation in the human brain [49-51]. As noted in the introduction, senile plaques occur preferentially near cerebral vasculature in AD human brain [33-36], as do dystrophic neurites associated with compact-plaques [44].

Using GFAP as a marker, we detected fairly robust astrocytic activation in the embolized cerebrum around the superficial cortex as well as in the white matter (wherein a low amount of GFAP IR is present under normal conditions). Notably, the increased GFAP IR occurred along the intracortical blood vessels invading the cortex from the pia. Densitometric data revealed an overall increase in GFAP IR in the cortex of embolized brains relative to sham-operated controls. These experimental findings are consistent with the notion that microvascular injury could induce glial inflammatory responses in the human brain [49-51].

Compared to the astrocytic changes, neuronal responses to experimentally induced microembolism primarily involved their terminals and processes around blood vessels which manifested as neuritic sphericles, sprouts and clusters. Small perivascular neuritic swellings were readily visible with NADPH-d histochemistry as early as 1 week post-embolization, while clusters of overtly swollen neurites developed around intracerebral blood vessels 2 and 4 weeks postlesion. These perivascular swollen neurites were immunoreactive for several neuronal phenotypic or transmitter markers including NADPH-d, PV, VGLUT-1 and ChAT. These results imply that with the current experimental paradigm used, microvascular injury can act as a causal factor to sufficiently induce focal dystrophic-like neuritic changes involving cortical glutamatergic principal cells, GABAergic interneurons, and putative forebrain cholinergic neurons. Notably, in AD human brain dystrophic neurites from different neuronal phenotypes may innervate the same plaque [52-54], while neuritic pathology can also occur in the absence of amyloid deposition [55, 56].

### Microembolism-induced neuritic pathology is associated with amyloidogenic potential

Many therapeutical agents developed thus far to enhance Aβ clearance from the brain (e.g., bapineuzumab, solanezumab, and most recently, gantenerumab) have failed at late-stage of clinical trials among individuals with mild or established AD, while these agents show promising efficacy in preclinical studies on transgenic AD models. The disappointing outcome of these human studies may be attributable to a slow but long pre-clinical course of brain Aβ elevation which impacts neuronal function to an extent that can no longer be rescued with Aβ immunization. These failed drug trials have also opened new discussion about the role of amyloid deposition in AD pathogenesis [30-32], and thus demonstrate the need for more basic studies to better understand the driving factor(s) of brain amyloidosis.

Our present study extends histological evidence for increased APP and BACE1 expression associated with neuritic pathology following experimentally induced cerebral microembolism in a natural/control animal model. The increased BACE1 labeling occurred largely in neuritic elements surrounding vasculature in the embolized cerebrum, with some large profiles colocalized with iron deposition indicative of vascular leakage. Increased neuritic APP labeling was also evident in the embolized cerebrum, including in the white matter, clearly suggesting the occurrence of axonal pathology. Extracellular Aβ deposition was detectable around some sites with a large amount of BACE1 or APP labeled elements. The finding of 6E10 colabeling in BACE1 immunoreactive neurites could imply an intraneuronal accumulation of β-CTF [27, 57], although it could also be related to intraneuronal APP or Aβ [58, 59]. Regardless of which is likely occurring, these abnormal neurites could represent a source for site-specific Aβ overproduction.

In summary, the present study uses guinea pigs and artificial insoluble carbon-based emboli to develop a histologically verifiable model of cerebral microembolism. We show clear anatomic evidence that experimental microembolism can induce vascular injury and leakage that is associated with glial inflammatory response and localized neuritic pathology. The neuritic alterations involve glutamatergic, GABAergic and cholinergic neurons, and are associated with increased immunolabeling of APP and BACE1, suggesting an enhanced amyloidogenic potential. Overall, the results are parsimonious with the notion that chronic microvascular injury could serve as a causal factor for neuroinflammation and AD-like neuritic pathology in the human brain.

## MATERIALS AND METHODS

### Ethics statement

Animal use was in accordance with the *National Institute of Health Guide for the Care and Use of Laboratory Animals*. All experimental procedures were approved by the Ethics Committee of Central South University Xiangya School of Medicine for animal care and use. Every effort was made to minimize stress and pain, and to avoid unnecessary use of experimental animals.

### Animals and experimental modeling of cerebral microembolism

A total of 70 Hartley guinea pigs aged 6 months were obtained from the animal center of Xiangya School of Medicine, and used for pilot and formal experiments in the present study. Unilateral common carotid artery infusion of coal particles in sterile saline was used as the experimental procedure to induce cerebral vascular microembolism. A series of pilot experiments were performed, involving infusion with particles ranging from 25-300 mesh-sizes for various time periods, followed by assessment of the resulted extent of cerebral vascular embolization. An optimal modeling approach was established by infusion of 1% (w/v, in saline) particles at 50-100 mesh-size (13-21 μm in diameter) between 5 to 10 minutes at a constant speed of 2 ml per minute, using a 20G needle propelled by an automatic pumping device (Model #WZL-506, Smiths Medical, CA, USA) placed about 30 mm higher than the surface of the operation table. Brains used for histological, histochemical or immunohistochemical examinations were from animals surviving 4 (n=8), 7 (n=4), 14 (n=4) and 30 (n=4) days after infusion with the above optimized procedure for 10 (surviving for 4 days) and 5 (surviving for 4-30 days) minutes. Additional animals were given the same cervical surgery excluding artery infusion (n=4/group, surviving 14 and 30 days). All sham-operated animals recovered from the surgery and survived until brain examination.

### Tissue preparation

The extent of in situ embolization was examined in the brains collected without perfusion from animals surviving 4 days. These animals were deeply anesthetized with sodium pentobarbital (100 mg/kg, i.p.), with the brains removed and photographed under a surgical scope, and then fixed by immersion in 4% paraformaldehyde in 0.01 M phosphate-buffered saline (pH 7.4, PBS) for 4 days. Other experimental and control animals were perfused via the ascending aorta with a vascular rinse (saline) followed by 4% paraformaldehyde in PBS, under deep anesthesia (100 mg/kg, i.p.). Brains were dissected out, postfixed in the perfusion fixative overnight at 4 °C. All brains were immersed in 30% sucrose for cryoprotection before cryostat sectioning. The cerebrum was cut at the frontal plane at 30 μm thickness, with 24 sets of sections collected serially in PBS in cell culture plates or by thaw-mounting directly on glass microslides. Sections were stored in a cryoprotectant at 20 °C until being used for batch-processing with histological, histochemical or immunohistochemical procedures.

### NADPH-diaphorase histochemistry

Sections from experimental and control brains were processed under identical conditions by incubation in 0.05 M Tris-HCl buffered saline (pH 8.0, TBS) containing 0.3% Triton X-100, 1 mM β-NADPH-d (Sigma-Aldrich, St. Louis, MO, USA; N7505), 0.8 mM nitroblue tetrazolium (NBT) (Sigma-Aldrich, N6639) and 5% dimethyl sulfoxide for 45 minutes at 37 °C. The histochemical reaction was stopped by rinsing the sections in PBS. The sections were then either mounted for microscopic examination of single NADPH-d labeling, or were undergone further immunohistochemical processing for other antigen markers using DAB as a chromogen.

### Immunohistochemistry

The primary antibodies used in the present study included: (1) monoclonal mouse anti-Aβ1-16, 6E10 (Signet, #39320, 1:4000); (2) mouse anti-APP (22C11) (EMD Millipore, MAB348, 1:2000); (3) rabbit anti-guinea pig albumin (BioSun Sci & Tech Co., Ltd, Shanghai, China); (4) rabbit anti-human BACE1 (1:2000) [26, 28, 60]; (5) goat anti-ChAT (EMD Millipore, AB1447, 1:1000); (6) rabbit anti-GFAP (Sigma-Aldrich, G9269, 1:4000); (7) mouse anti-VGLUT1 (EMD Millipore, MAB5502, 1:2000), and (8) mouse anti-parvalbumin (Sigma-Aldrich, G9269, 1:4000). All these antibodies have been widely used in research and reported in literature.

For immunolabeling with the peroxidase-DAB method, unstained sections, together in some cases with the ones went through the above-described NADPH-d histochemistry, were treated with 3% H_2_O_2_ in PBS for 30 minutes to deactivate endogenous peroxidase, followed by a pre-incubation in 5% normal horse serum with 0.3% Triton X-100 for 1 hour to lower nonspecific antibody binding. For BACE1 and Aβ antibody staining, the sections were also treated in 50% formamide and 50% 2SSC at 65 °C for 1 hour and 50% formic acid in PBS for 30 minutes at room temperature, respectively. The sections were subsequently incubated overnight at 4 °C with a primary antibody diluted in PBS containing 5% normal horse serum. Next the sections were reacted with a biotinylated pan-specific secondary antibody at 1:400 for 2 hours, and further with the ABC reagents (1:400) (Vector Laboratories, Burlingame, CA, USA) for an additional hour. Immunoreactivity was visualized using 0.003% H_2_O_2_ and 0.05% diaminobenzidine (DAB, Sigma-Aldrich).

For double immunofluorescence, sections from embolized and sham-operated animals were incubated in PBS containing 5% donkey serum for 1 hour, and then in the same buffer containing a pair of primary antibodies from different animal species for 12 hours at 4°C. The sections were further brought to react for 2 hours with Alexa Fluor® 488 and Alexa Fluor® 594 conjugated donkey anti-mouse, rabbit or goat IgGs (1:200, Invitrogen). The sections were counterstained with bisbenzimide before being mounted with anti-fading medium.

### Imaging analysis

Brain sections were examined and imaged on an Olympus microscope (BX53, CellSens Standard, Olympus, Japan) and a confocal microscope (Nikon, DIGITAL ECLIPSE C1 plus). Quantitative image analyses were carried out using Image-J (NIH) or the OptiQuant (Parkard Instruments, Meriden, CT, USA) software. For comparative densitometry, sections passing four landmark brain structures, i.e., the anterior commissure, the mid-septum, the anterior end of hippocampus and the temporal pole, were selected for imaging. The parietotemporal neocortical regions, including the cortex and white matter, in these sections were imaged with the 10× objective lens, using an identical exposure setting for all comparing brains. Following image documentation, vascular profiles filled with the infused coal particles were automatically selected using a threshold obtained at an area that did not contain any embolized vessel. The areas covered by the embolized vascular profiles as well as the total image area were reported. The ratio (%) of area occupied by the embolized vessels in each animal was then calculated. For measuring the optic density of immunoreactivity [expressed as digital light units per square millimeter (DLU/mm^2^)], the area of interest (AOI) was set to layers I-VI of the parietotemporal neocortex at the above-mentioned cortical levels. A specially designed quantification method was used to determine the extent of embolization to leptomeningeal arteries, using the images taken at brain removal. This was achieved by counting the numbers of embolized and non-embolized pial arterial sites intersecting at each of the vertical and horizontal lines of a 10×10 grid measuring template (illustrated in Fig. 1C). The template was set to have a total width equal to the distance between the mid-sagittal fissure to the temporal pole (i.e., the widest part of the cerebrum), and was placed over the image of the dorsal cerebral surface on each hemisphere, with its horizontal half-dividing line overlapped on a line connecting the temporal poles.

### Statistical testing and figure preparation

Numerical and densitometric data were expressed as mean±standard derivation (SD). Statistical comparison of means was performed using two-tailed Student *t*-test (Prism GraphPad 4.1, San Diego, CA). The minimal significant level of difference between means was set at P<0.05. Graphic and section images were assembled with Photoshop 7.1.
